# Genetic Case Control Analysis of the Multi‐Partner Consortium to Expand Dementia Research in Latin America (ReDLat) Cohort

**DOI:** 10.1002/alz70861_108949

**Published:** 2025-12-23

**Authors:** J. Nicholas Cochran, Jared W Taylor, Juliana Acosta‐Uribe, Stefanie D Pina‐Escudero, Patricia A. Castruita, Caroline Jonson, Erin A. Barinaga, Sydney Quinn Johnston, Mike A Nalls, Dan Vitale, José Alberto Avila Funes, María Isabel Behrens, Martin Alejandro Bruno, Luis Ignacio Brusco, Nilton Custodio, Claudia Duran‐Aniotz, Francisco Lopera, David Aguillon, Diana L Matallana, Andrea Slachevsky Chonchol, Leonel Tadao Takada, Lina Maria Zapata‐Restrepo, Bruce L. Miller, Victor Valcour, Agustin Ibanez, Ken Kosik, Jennifer Yokoyama, the Multi‐Partner Consortium to Expand Dementia Research in Latin America (ReDLat)

**Affiliations:** ^1^ HudsonAlpha Institute for Biotechnology, Huntsville, AL USA; ^2^ University of California Santa Barbara, Santa Barbara, CA USA; ^3^ Grupo de Neurociencias de Antioquia, Universidad de Antioquia, Medellin, Medellin Colombia; ^4^ Memory and Aging Center, UCSF Weill Institute for Neurosciences, University of California, San Francisco, San Francisco, CA USA; ^5^ Global Brain Health Institute, San Francisco, CA USA; ^6^ Memory and Aging Center, Department of Neurology, Weill Institute for Neurosciences, University of California, San Francisco, San Francisco, CA USA; ^7^ University of California, San Francisco, San Francisco, CA USA; ^8^ National Institute on Aging, Bethesda, MD USA; ^9^ Data Tecnica International, Washington, DC USA; ^10^ Instituto Nacional de Ciencias Médicas y Nutrición Salvador Zubirán, México, DF Mexico; ^11^ Departamento de Neurología y Neurocirugía, Hospital Clínico, Universidad de Chile., Santiago, Región Metropolitana de Santiago Chile; ^12^ Instituto de Ciencias Biomédicas (ICBM) Facultad de Ciencias Médicas, Universidad Catoóica de Cuyo, San Juan, San Juan Argentina; ^13^ National Scientific and Technical Research Council (CONICET), Buenos Aires Argentina; ^14^ Unit Cognitive Impairment and Dementia Prevention, Peruvian Institute of Neurosciences, Lima, Peru, Lima, Lima Peru; ^15^ Geroscience Center for Brain Health and Metabolism (GERO), Santiago Chile; ^16^ Neurosciences Group of Antioquia, University of Antioquia, Medellin Colombia; ^17^ Grupo de Neurociencias de Antioquia, Facultad de Medicina, Universidad de Antioquia, Medellín Colombia; ^18^ Hospital Universitario San Ignacio, Bogotá, Bogotá Colombia; ^19^ Associação Revivendo Memórias, São Paulo, São Paulo Brazil; ^20^ Facultad de Ciencias de la Salud, Universidad Icesi, Cali, Colombia, Cali Colombia; ^21^ Global Brain Health Institute, University of California, San Francisco, San Francisco, CA USA; ^22^ Global Brain Health Institute (GBHI), Trinity College Dublin (TCD), Dublin, Dublin Ireland; ^23^ Universidad de San Andrés, Buenos Aires Argentina; ^24^ University of California, Santa Barbara, Santa Barbara, CA USA

## Abstract

**Background:**

Genetic studies for Alzheimer's disease and related dementias are underrepresented in Latin America. The Multi‐Partner Consortium to Expand Dementia Research in Latin America (ReDLat) cohort recruits from ten centers in six countries across Latin America, providing an opportunity to address this shortcoming.

**Method:**

2,736 participants with available genetic material were assessed via Illumina genome sequencing. Genetic ancestry was determined via the package GenoTools and data were analyzed separately by genetic ancestry group. The largest genetic ancestry group was admixed American (AMR) with 2,147 individuals. Of these 2,147 individuals, 65 were excluded in quality control steps leaving 981 controls, 771 AD, 298 FTD, and 32 with other or indeterminate phenotypes. GWAS was conducted using GEMMA which adjusts for admixture and familial relationships using a kinship matrix. Burden analysis was also conducted with adjustment via a kinship matrix.

**Result:**

In the AD GWAS, the only genome‐wide significant (*p* < 5x10^‐8^) hit in preliminary analysis was the *APOE* locus, with rs429358 (which tags the *APOE* ε4 allele) as the lead snv with *p* = 8x10^‐42^. 103 independent loci (defined by separation by at least 1 MB) exhibited suggestive significance (*p* < 1x10^‐5^), with 7 of these beyond the APOE locus exhibiting replication with at least *p* < 0.05 and consistent direction betas for the effect allele in Bellenguez, et al. 2022 summary statistics. These loci included rs113181288 on chromosome 1 Between *ELF3* and *ENSG00000294430*, rs4971744 on chromosome 2 (*NRXN1‐DT* intronic), rs76343365 on chromosome 4 between *ENSG00000289869* and *ENSG00000250945*, rs7442695 on chromosome 5 (*ENSG00000294529* intronic), rs62437151 on chromosome 6 (*ENSG00000223786* intronic), rs117134121 on chromosome 10 (*ADARB2* Intronic), and rs2278745 on chromosome 10 (*HK1* Intronic). GWAS for FTD and burden analyses for AD and FTD are underway.

**Conclusion:**

Expansion of recruitment efforts for genetic studies in ADRD has great potential to nominate new hits for further study. This analysis is an important step forward for representation of Latin Americans in genetic association studies for ADRD.

